# Untangling the Gordian Knot of *Aplysia* sea hare egg masses: An integrated open-hardware system for standardized egg strand sizing and packaging for cryopreservation research and application

**DOI:** 10.1016/j.ohx.2023.e00476

**Published:** 2023-10-31

**Authors:** Jack C. Koch, Allyssa M. Oune, Sarah Bodenstein, Terrence R. Tiersch

**Affiliations:** a Aquatic Germplasm and Genetic Resources Center (AGGRC), School of Renewable Natural Resources, Louisiana State University Agricultural Center, Baton Rouge, LA 70820, United States

**Keywords:** Marine invertebrate cryopreservation, Hardware-augmented protocol, Germplasm repositories, Genetic resources, *Aplysia californica*, 3-D printing

## Abstract

The California sea hare (*Aplysia californica*) provides a powerful biomedical model system for studying aspects of neurological development and damage, behavior, aging, and hypoxia. *Aplysia* encapsulate their zygotes within strands that result in tangled egg masses that greatly complicate culture and experimentation. The historical and current importance of *Aplysia* for biomedical research and the mounting climate crisis necessitates protection of *Aplysia* genetic resources. The goal of this work was to prototype open-hardware sizing, processing, and packaging devices for *A. californica* early life stages suitable for integration into a cryopreservation pathway. The Strand Centi-Sizer was a low-cost, fused filament fabrication 3-D printable device that increased experiment preparation efficiency and standardized the cutting of egg strands customizable to user needs. A downstream system of 3-D printed devices was also prototyped to address inefficiencies in handling of egg strand sections for processing and packaging into existing cryopreservation straw platforms. Time studies were conducted comparing manual methods (i.e., no specialized equipment) with open hardware to demonstrate utility of the devices and to encourage community members to design and prototype new devices to address recurrent and novel problems in other aquatic animals that produce egg strands. Improvements in design could further increase efficiency, standardization, and reproducibility, and extend the application of these devices to other research communities, such as shrimp or salamander spermatophores, sea anemone body part (e.g., pedal lacerate) cryopreservation, or study areas such as vitrification.

## Graphical abstract

**Figure:**
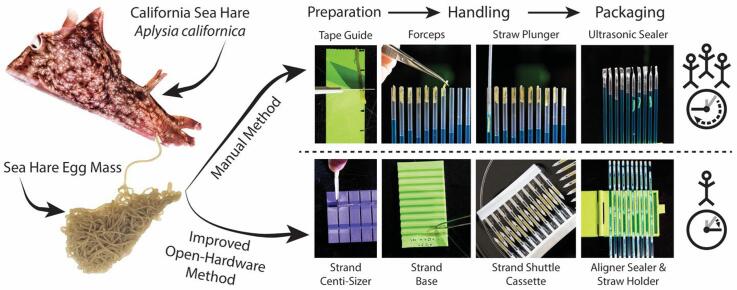

**Table:** 

Specifications table	
Hardware name	Strand Cryopreservation Shuttle System (SCSS)
Subject area	Biological sciences
Hardware type	Biological sample handling and preparation
Closest commercial analog	IMV Technologies Cryo Bio Systems (CBS) High Security Vitrification Straw (https://www.imv-technologies.in/product/cbs-high-security-vitrification-straw)
Open-source license	Creative Commons Attribution 4.0 International (CC BY NC SA 4.0)
Cost of hardware	Material cost per Strand Cryopreservation Shuttle System: US$2.14
Source file repository	http://doi.org/10.5281/zenodo.8033755 https://3d.nih.gov/entries/3DPX-020446

## Hardware in context

The safeguarding of economically relevant agricultural species has historically been accomplished by storing, evaluating, and distributing genetic resources as cryopreserved germplasm maintained in repositories. These repositories have been part of a multi-billion-dollar industry for livestock since the early 1960s. The shift to develop cryopreservation repositories and devices has been slow within the broader scientific community, especially for aquatic species such as marine invertebrates despite the global economic value that they generate annually [1], [2], [3] and the dire need to protect imperiled aquatic species [4], [5], [6]. Initiatives to build repositories for marine invertebrates could begin with model organism communities [7]. These organisms are well studied and have research communities with vested interests in protecting the genetic resources being developed through billions of dollars in research grants.

The California sea hare (*Aplysia californica*) is a biomedical model organism used primarily to study neurological development and damage [8], behavior (i.e., learning and memory, [9], [10], [11]), aging [12], and hypoxia tolerance. These sea hares are recognized for their well characterized nervous system, massive neurons, and simple life cycle. Hundreds of millions of research dollars are distributed annually for studies that make use of *Aplysia*. The *Aplysia* research community relies on the services and animal husbandry of staff at the National Resource for *Aplysia* (NRA, University of Miami) to provide all life stages of *Aplysia* to accomplish this research. Husbandry processes are cost and labor intensive, requiring a supply of chilled seawater, large quantities of macroalgae, and ample space for growing of animals to the various stages and sizes required by the community. The NRA reproduces some animals in-house but due to complications with inbreeding, relies on semi-annual shipments of wild animals to replenish genetic diversity. Shipments of wild animals have some uncertainty as the understanding of wild populations is limited, animals can be unpredictable to locate, and as the number of wild animal collectors decline. These barriers paired with the power of these animals as a biomedical model necessitate development of a germplasm repository and cryopreservation pathway to protect the research investments made in *Aplysia.* In addition, repository storage of frozen material will provide new opportunities for the research community to preserve genetic diversity and to create and maintain mutant and transgenic lines with reduced cost, resources, and risk. The broader intention of developing a cryopreservation pathway for a model organism such as *Aplysia* is to extend the pathway to minor and emerging model organisms, and other aquatic invertebrates such as oysters and imperiled corals.

To date there are no published cryopreservation pathways for *Aplysia* sea hares. In addition, there are multiple cryopreservation challenges related to aspects of *Aplysia* reproduction and spawning. For example, *Aplysia* are simultaneous, non-self-fertilizing hermaphrodites that perform internal fertilization [13]. With the current understanding of this reproductive strategy and available technologies, obtaining sperm from *Aplysia* is inefficient and impractical. Thus, cryopreservation efforts have been focused on early life stages including embryos and larvae which are more challenging to work with.

**Fig. 1:**
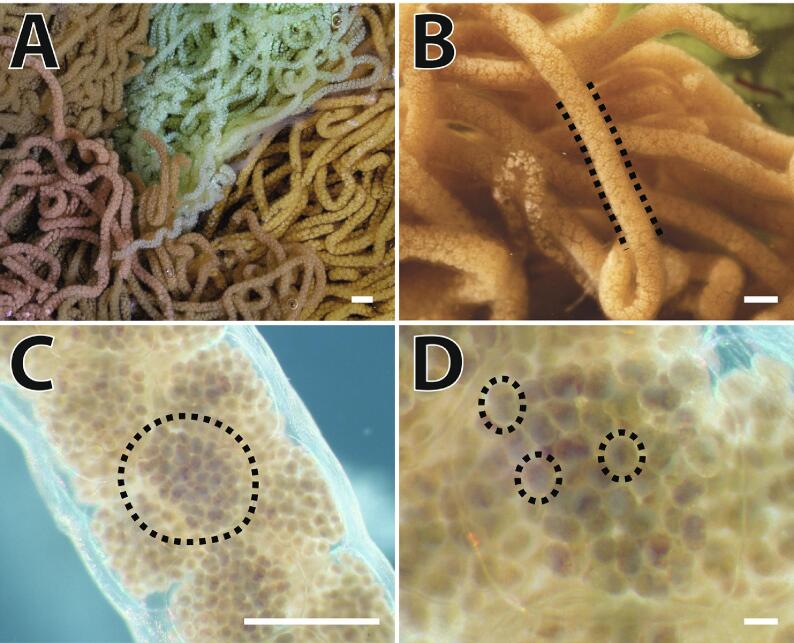
Overview of *Aplysia* egg masses. Egg masses are deposited in a mass of tangled strands (A, 3 mm), often from multiple animals, and the strands (B, dotted, 1.5 mm) contain concatenated capsules arranged helically (C, dotted, 750 µm). Individual embryos (15–100) and larvae (D, dotted, 40 µm) are enclosed within each capsule.

Like many other groups of marine invertebrates, the early life stages of *Aplysia* are packaged within egg capsules, helically organized within tangled strands that are deposited as sticky egg masses, hardening shortly after being deposited (Fig. 1). *Aplysia* egg masses are quite large and are very complicated compared to sperm cells. Masses are approximately the size of a baseball, weigh as much as 70 g, and can contain hundreds of millions of embryos. In addition to size, the layers of organization within an egg mass present challenges during cryopreservation including handling, preparation, packaging, and storage. Because most cryopreservation technologies are intended for use with single cells such as sperm, they are not suitable for *Aplysia* egg masses or strands. In addition, cryopreservation has commercial-scale equipment and platforms to support high-throughput and quality management practices that contribute to the success of established industries. However, even if useful for *Aplysia*, these devices are prohibitively expensive. Therefore, this challenge is more like cryopreserving a tissue or organ rather than cells, and new ideas and devices are required. Open-hardware devices are a promising option to simultaneously overcome these challenges as they can be accessed by a wide variety of community members, rapidly prototyped and customized based on user needs, and can be integrated into existing protocols and procedures [14].

Device creation must consider the container that will hold the cryopreserved samples (e.g., French straw, cryovial). Container choice is the first and most important aspect in developing a cryopreservation pathway. The sample container dictates the size and shape of novel accessory devices, what existing platforms and technologies are available to load, seal, label, and store samples, the cooling rate that can be achieved, how many samples can be placed in cryogenic storage, and other cryopreservation factors [15]. Planning for integration of new devices into a widely used storage container (i.e., 0.5-mL and 0.25-mL French straws) will greatly increase the likelihood of a community adopting cryopreservation. Furthermore, developing accessories for an established freezing container ensures there will be resources available to increase efficiency and scale of the pathway [16], [17].

The handling, preparation, packaging, and storage of *Aplysia* egg masses are critical steps, each presenting unique challenges. Necessary considerations were reduction of the size of the egg mass to be suitable for cryopreservation, packaging into existing cryopreservation containers, and being adoptable for the *Aplysia*-culture community. To address these needs, an innovative family of open-hardware three-dimensional (3-D) printed devices was developed called the Strand Cryopreservation Shuttle System (SCSS) that accomplishes strand sizing with the Strand Centi-Sizer (v4.0.1), and strand positioning and packaging with the Straw-Strand Shuttle System (4S). The 4S consisted of the Strand Shuttle Cassette (v6.1.16), Aligner Sealer (v2.1.4), Strand Base (v4.0.0), and Straw Holder (v1.8.6). The specific objectives were to: (1) design a device to section *Aplysia* egg strands into 1-cm pieces; (2) design a system of devices that could position, capture, and load the sized egg strand pieces into standard 0.5-mL French straws; (3) prototype devices suitable for 3-D printing with consumer-level printers, and (4) evaluate the feasibility, efficiency, and application of the devices through time studies. Overall, the devices and methods developed herein are useful not only for research and commercial application but also for basic discovery. This hardware will substantially increase efficiency as well as accelerate and improve cryopreservation research and application for the NRA, thereby supporting the overall research community. Open hardware will empower researchers working with minor and emerging models by providing a powerful foundation to support scientific research goals with a common standard.

## Hardware description

### Strand Centi-Sizer

**Fig. 2:**
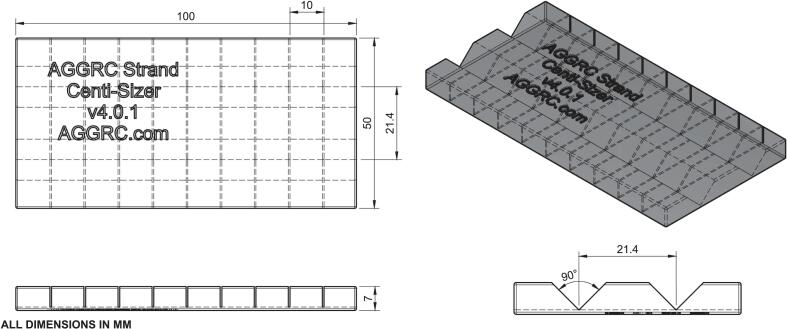
Schematic drawing of a Strand Centi-Sizer with 1-cm cutting intervals. Top view, isometric view, side view, end view (all dimensions in mm).

Prior to open-hardware device development, *Aplysia* egg masses were dissected into strands by use of a scalpel or scissors, measured to approximate size by eye or tape guide, and cut again by use of a scalpel or razor blade. The approach mirrored the traditional use of manual (i.e., no specialized hardware), self-limiting, solutions to research problems. This process was time consuming and required excessive person-hours (# of workers × amount of time per person to accomplish a task) depending on the complexity of an egg mass (e.g., how tightly it was wound) and how many strand pieces were required. The Strand Centi-Sizer was developed to increase efficiency and accuracy of cutting *Aplysia* egg strands in precisely sized, 1-cm pieces. The Strand Centi-Sizer was printed by use of a fused filament fabrication (FFF) 3-D printer (Table 2, Table 3) with polylactic acid (PLA) thermoplastic filament (ZYLtech, Houston, Texas). The device was 50 mm × 120 mm × 7 mm and had two parallel guide grooves (spaced 21.4 mm on center) and multiple cutting channels, perpendicular to the grooves, spaced according to the length of egg strand required by the user (Fig. 2). For the time studies herein, 1-cm channel spacings were assessed. The width of the cutting channels could accommodate a scalpel or razor blade and the distance between them was customizable through edits in computer-aided design (CAD) software (Fusion360, Autodesk, San Francisco, California). A single bar could accommodate multiple channel spacings (e.g., 6, 1-cm channels and 16, 0.25-cm channels on the same bar) to increase the range of applications.

The ease of use and reliability of the Strand Centi-Sizer were significant improvements compared to other methods such as the tape guide. The approximate material cost of a Strand Centi-Sizer that cut 1-cm pieces was estimated to be US$0.80 (40 g of PLA) compared to a plastic ruler (>US$0.50) and a comparably sized piece of laboratory tape (∼US$0.06). The Strand Centi-Sizer was reusable, much like a ruler, and was more durable compared to tape. Ease of use was high for the Strand Centi-Sizer as the measurements between cutting channels were identical, precise, and set. There was no need to mark measurements that could be inaccurate or inconsistent. The cutting grooves contributed to the ease of use as they guided the blade during the cutting motion rather than an unguided cut when using a tape guide. In addition, multiple strands could be placed in the parallel guide grooves simultaneously to further increase efficiency.

### Straw-Strand Shuttle System (4S)

**Fig. 3:**
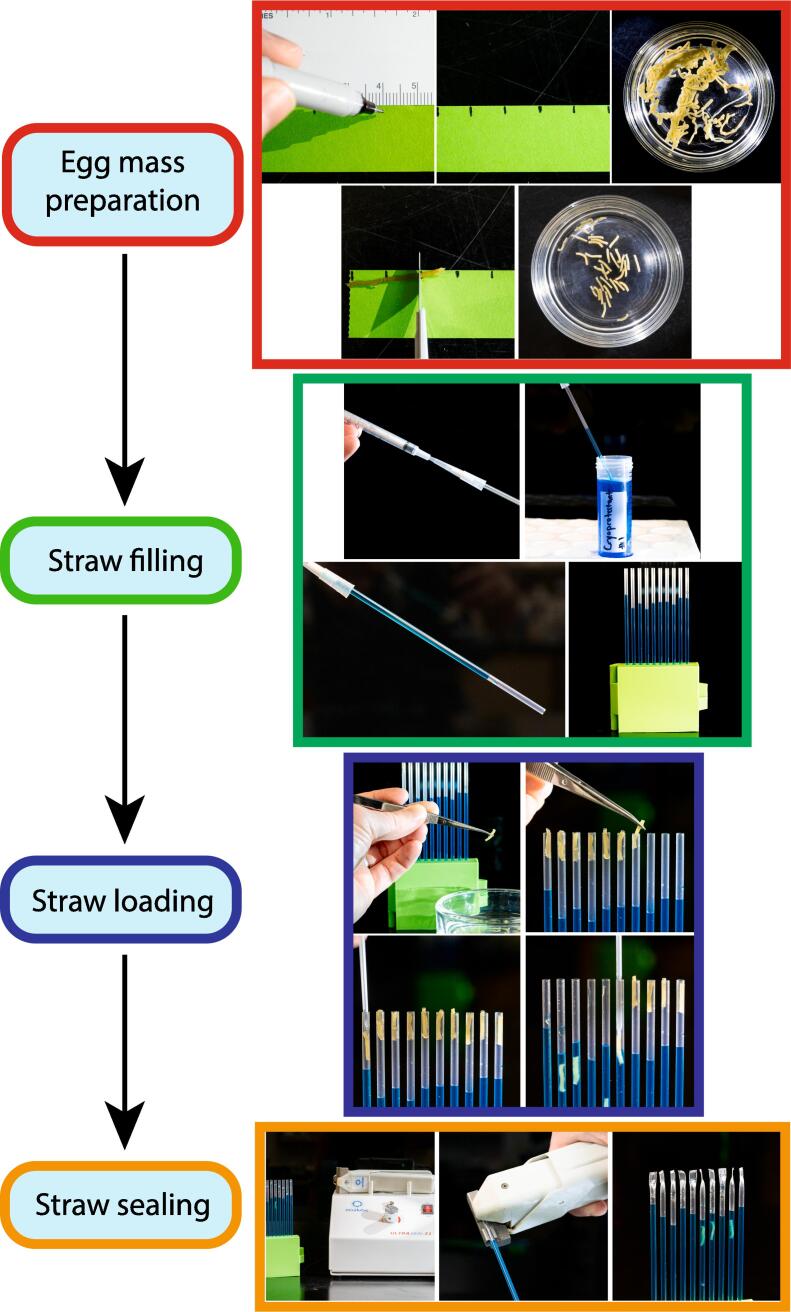
(Left) Simplified process map and (Right) timelapse images of the manual “load-and-plunge” method.

Handling and packaging egg strands in French straws for cryopreservation presented unique challenges. For example, handling egg strands after they were cut was difficult because they were small and could be crushed or damaged by excessive pressure with forceps. Use of forceps with a limit screw (>US$65) to prevent excess pressure was an option, but a reduced need for forceps by use of open hardware presented the advantages of customization and standardization. With initial manual approaches, packaging of egg strands for cryopreservation resembled a “load-and-plunge” technique where a sized egg strand was painstakingly inserted into the open end of 0.5-mL French straw using forceps. This was followed by pushing the strand piece into the cryoprotectant-filled straw by use of a smaller (0.25-mL) French straw, thus initiating an equilibration process (Fig. 3). This technique was quite variable, making reproducibility of outcomes challenging, and only allowed a single egg strand to be loaded per straw. In addition, this method sometimes resulted in the egg strand becoming lodged within the 0.25-mL “plunger” straw (this was amended by sealing one end of the 0.25-mL straw).

**Fig. 4:**
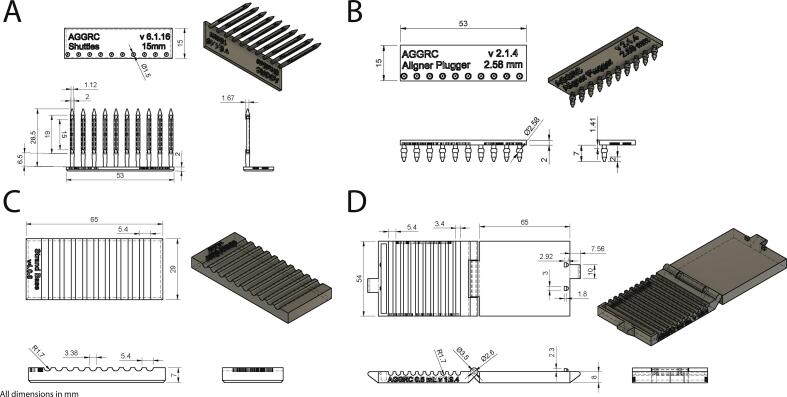
Schematic drawings of (A) Strand Shuttle Cassette, (B) Aligner Sealer, (C) Strand Base, and (D) Straw Holder. (A) Side view, isometric view, top view, end view; (B, C, and D) Top view, isometric view, side view, end view. All dimensions in mm.

The Straw-Strand Shuttle System (4S) was developed to increase the efficiency of preparing and packaging strands for cryopreservation, introduced reproducibility, and standardized handling. The 4S also accomplished a reduction in the need for forceps during strand handling. There were four components that comprised the 4S: Strand Shuttle Cassette (v6.1.16), Aligner Sealer (v2.1.4), Strand Base (v4.0.0), and Straw Holder (v1.8.6) (Fig. 4). The Strand Shuttle Cassette and Aligner Sealer were resin printed (Table 2, Table 3) with a custom mix of 2:1 basic resin (Anycubic, Shenzhen, China) to flexible resin (Monocure3D, Sydney, Australia). The Straw Holder and Strand Base were FFF printed (Table 2, Table 3) with PLA (ZYLtech, Houston, Texas).

### Strand Shuttle Cassette

The Strand Shuttle Cassette was designed to capture and support 10 egg strand pieces up to 1 cm in length for application to equilibrium cooling. Each cassette had a rectangular base (15 mm × 53 mm × 2 mm) with 10 perpendicular shuttles (Fig. 4A). Each shuttle was 28.5 mm × 2 mm allowing it to fit unimpeded within a 0.5-mL French straw. Shuttles were spaced 5.40 mm on center to match the straw spacing on most 0.5 mL-compatible cryopreservation equipment. Each shuttle was connected to the base by a tapered cylinder to facilitate removal of the shuttle from the cassette. Distal to the tapered cylinder was a cage-like slot (15 mm x 1.12 mm) designed to hold a single egg strand piece. The slot had an open back to reduce the thermal mass surrounding the egg strand and open ends to facilitate cryoprotectant solution equilibration and shuttle movement down the length of a French straw. The most distal end of each cassette was topped with a 3-mm 15° conical point to assist in guiding the shuttle into a straw. The number, size, and spacing of the shuttles were customizable through edits in CAD software (Fusion360, Autodesk, San Francisco, California). In this configuration a single 0.5-mL French straw could hold three shuttles. The estimated material cost of a Strand Shuttle Cassette was US$0.05 (1.90 mL of resin) or US$0.005 per shuttle.

### Aligner Sealer

The Aligner Sealer was designed to have two roles: align strands on the Strand Base and seal straws without needing heat, water (to moisten PVA sealing powder), or electrical power. The Aligner Sealer was a rectangular device (15 mm × 53 mm × 2 mm) with 10, 5-mm long cylindrical pegs (Fig. 4B). Each peg had a 2.58-mm diameter ball attached 1.4 mm from the base, and each was topped with a 2-mm 15˚ conical point to assist in guiding the peg into a straw. Peg spacing aligned with the grooves on the Strand Base and the straws in the Straw Holder. The size of the sealing ball and spacing of the pegs were customizable through edits in CAD software (Fusion360, Autodesk, San Francisco, California). The estimated material cost of an Aligner Sealer was US$0.05 (1.72 mL of resin) or US$0.005 per sealer.

### Strand Base

The Strand Base was designed to support egg strand pieces in preparation for loading into Strand Shuttle Cassette shuttles. The base was a rectangular device (29 mm × 65 mm × 7 mm) with 10 parallel grooves ∼3.2 mm wide and ∼1.8 mm deep to accommodate egg strands and shuttles (Fig. 4C). Groove spacing aligned with shuttles on the Strand Shuttle Cassette and pegs on the Aligner Sealer. The depth, width, and spacing of the grooves were customizable through edits in CAD software (Fusion360, Autodesk, San Francisco, California). The estimated material cost of a Strand Base was US$0.14 (7 g of filament).

### Straw Holder

The Straw Holder was designed to firmly hold 10, 0.5-mL French straws. The straw-holding side was a rectangle (54 mm × 65 mm × 8 mm) with 10 parallel grooves ∼3.4 mm wide and ∼1.9 mm deep (Fig. 4D). The pressure side was a rectangle (54 mm × 65 mm × 8 mm) with no grooves. The two side were connected by a hinge. There was a locking mechanism that enabled straws to be held in place to facilitate loading of shuttles and straw sealing. The depth, width, and spacing of the grooves were customizable through edits in CAD software (Fusion360, Autodesk, San Francisco, California). The estimated material cost of a Straw Holder was US$0.58 (29 g of filament).

The cost of the 4S was low compared to similar hardware like the IMV CBS High Security Vitrification Straws. The approximate total material cost of a 4S package (3 Strand Straw Cassettes, 1 Aligner Sealer, 1 Strand Base, and 1 Straw Holder) was estimated to be US$1.34 compared to US$64 for a 4-pack of IMV CBS High Security Vitrification Straws. In addition to cost, the 4S was highly customizable compared to other hardware.

These devices could benefit other users in multiple ways including:

• Enabling materials to be cut simultaneously (with use of parallel guide grooves) to standardized lengths (Strand Centi-Sizer).
• Capturing and handling fragile or small materials with reduced human input (4S).
• Assisting in the loading of samples into French straws or other containers with permanent labeling and secure storage options (4S).
• Application in vitrification of egg strands, spermatophores, sperm, or other germplasm types (4S).

## Design files summary

### Design files

Table 1.

**Table 1:** File descriptions.

Design file name	File types	Open-source license	Location of the files
AGGRC Strand Centi-Sizer v4.0.1 (Parametric Uniform 1-cm Cut)	F3D, 3MF, STEP, STL	Creative Commons Attribution 4.0 International (CC BY NC SA 4.0)	http://doi.org/10.5281/zenodo.8033755
AGGRC Strand Centi-Sizer v4.0.1 (Parametric Mixed Cut)	F3D, 3MF, STEP, STL	Creative Commons Attribution 4.0 International (CC BY NC SA 4.0)	http://doi.org/10.5281/zenodo.8033755
AGGRC Strand Shuttle Cassette v6.1.16 and Aligner Sealer v2.1.4	F3D, 3MF, STEP, STL	Creative Commons Attribution 4.0 International (CC BY NC SA 4.0)	http://doi.org/10.5281/zenodo.8033755
AGGRC Strand Base v4.0.0	F3D, 3MF, STEP, STL	Creative Commons Attribution 4.0 International (CC BY NC SA 4.0)	http://doi.org/10.5281/zenodo.8033755
AGGRC Straw Holder v1.8.6	F3D, 3MF, STEP, STL	Creative Commons Attribution 4.0 International (CC BY NC SA 4.0)	http://doi.org/10.5281/zenodo.8033755

### Bill of materials

In the present study the filament cost was US$20 per kg (ZYLtech, Houston, Texas), the basic resin cost was US$69 per L (Anycubic, Shenzhen, China), and the flexible resin cost was US$80 per L (Monocure3D, Sydney, Australia). Thus, the material costs were estimated at US$0.02 per g for FFF components and US$0.08 per mL for resin components. For FFF printing, the mass of filament and printing time were estimated in Ultimaker Cura (v5.2.1, Ultimaker, Utrecht, Netherlands) and for resin printing, the mass of resin and printing time were estimated in Chitubox Basic (v1.8.1, Chitubox, Guangdong, China).

## Bill of materials summary

Table 2

**Table 2:** Bill of materials.

Designator	Component	Unit	Cost (US$) per unit	Total cost (US$)	Print Time (Printer)	Material type
AGGRC Strand Centi-Sizer v4.0.1 (Parametric Uniform 1-cm Cut)	Centi-Sizer (1 cm)	33 (g)	0.02	0.66	4 hr 12 min (SOVOL SV01)	PLA
AGGRC Strand Centi-Sizer v4.0.1 (Parametric Mixed Cut)	Centi-Sizer (1 cm and 0.25 cm)	40 (g)	0.02	0.80	5 hr 2 min (SOVOL SV01)	PLA
Disposable Scalpel	Scalpel	1	0.83	0.83	–	Plastic and stainless steel
Forceps	Forceps	1	1.97	1.97	–	Stainless steel
**Strand Centi-Sizer Total**	–	<40 (g)	–	3.60	<5 hr 2 min	–
AGGRC Strand Shuttle Cassette v6.1.16	Strand Shuttle Cassette	1.90 (mL)	0.08	0.16	3 hr (Phrozen Sonic Mini)	2:1 Basic Resin:Flexible Resin
AGGRC Aligner Sealer v2.1.4	Aligner Sealer	1.72 (mL)	0.08	0.14	55 min (Phrozen Sonic Mini)	2:1 Basic Resin:Flexible Resin
AGGRC Strand Base v4.0.0	Strand Base	7 (g)	0.02	0.14	49 min (SOVOL SV01)	PLA
AGGRC Straw Holder v1.8.6	Straw Holder	29 (g)	0.02	0.58	2 hr 52 min (SOVOL SV01)	PLA
Straw-Strand Shuttle System (4S) Total*	–	36 (g) 7.42 (mL)	–	1.34	6 hr 40 min**	–
Strand Cryopreservation Shuttle System (SCSS) Total***	–	76 (g) 7.42 (mL)	–	4.94	11 hr 42 min	–

*Includes 3 Strand Straw Cassettes, 1 Aligner Sealer, 1 Strand Base, and 1 Straw Holder.

**Printing 1 Aligner Sealer and 3 Strand Shuttle Cassettes on one build plate.

***Includes average cost of a Strand Centi-Sizer and a 4S package.

## Build instructions

### Strand Centi-Sizer

1) Print Strand Centi-Sizer with the desired cutting channel spacing. The slicing settings for Ultimaker Cura (v5.2.1, Ultimaker, Utrecht, Netherlands) are provided in Table 3.

**Table 3:** Specifications for 3-D printing and post processing of the Strand Cryopreservation Shuttle System (SCSS).

Parameters	Specifications	Parameters	Specifications
Components	Strand Base and Straw Holder	Components	Strand Shuttle Cassette and Aligner Sealer
Slicing software	Ultimaker Cura	Slicing software	Chitubox Basic
Software version	5.2.1	Software version	1.8.1
Filament manufacturer	ZYLtech Engineering	Hard resin manufacturer	AnyCubic
Material	Polylactic acid	Hard resin	Basic
Filament diameter	1.75 mm	Flexible resin manufacturer	Monocure 3D
Printer	SOVOL SV01	Flexible resin	Flex100
Printer firmware	1.1.6.1	Printer	Phrozen Sonic Mini
Layer height	0.15 mm	Printer firmware	1.3.7
Initial layer height	0.3 mm	Layer Height	0.050 mm
Wall thickness	0.8 mm	Bottom layer count	6
Wall line count	3	Bottom layer exposure time	35 s
Top surface skin layers	3	Transition layer count	0
Top/Bottom thickness	0.8 mm	Exposure time	8 s
Top layers	4	Rest time before lift	2 s
Bottom layers	3	Rest time after lift	0 s
Infill density	20 %	Rest time before retract	2 s
Infill pattern	Octet	Bottom lift distance	6 mm
Printing temperature	200˚C	Lifting distance	
Printing temperature initial layer	215˚C		
Build plate temperature	60˚C		
Build surface material	Textured glass surface	Bottom lift speed	60 mm/min
Print speed	50 mm/s	Lifting speed	
Infill speed	150 mm/s	150 mm/min	
Wall speed	100 mm/s		Retract speed
Top/Bottom speed	50 mm/s	Number of > 70 % isopropyl alcohol washes	2
Initial layer speed	40 mm/s	Wash time	At least 4 min
Number of slower layers	3	Dry time	At least 30 min
Flow equalization ratio	100 %	Cure time	5 min
Retraction	Enabled		
Print cooling	Enabled		
Fan speed	100 %		
Build plate adhesion type	Skirt

### Straw-Strand Shuttle System (4S)

1) Print Straw Holder and Strand Base with appropriate settings (Table 3). In the present study these were printed separately, but it is possible to print them on the one build plate at the same time.
2) Print Strand Shuttle Cassettes and Aligner Sealer. The slicing settings for Chitubox Basic (v1.8.1, Chitubox, Guangdong, China) and the suggested post-processing procedures are provided in Table 3.

## Operation instructions

### Strand Centi-Sizer (Fig. 5)

While performing this procedure, wear appropriate personal protective equipment, including eye protection, laboratory coat, and protective gloves. Use caution when using a scalpel. Moisten the Strand Centi-Sizer by dipping it in artificial seawater (28–32 ppt) before laying it on a flat surface with the cutting channels facing up. Place an untangled egg strand in the long, horizontal guide channel in alignment with one end of the Strand Centi-Sizer. Draw a scalpel through each vertical cutting channel to cut the egg strand at the specified length intervals. Take caution to avoid cutting the fingers of the operator when the scalpel is entering and exiting each cutting channel. After the egg strand is cut, place one end of the Strand Centi-Sizer over a glass bowl filled with artificial seawater. Draw a medium-tipped forceps down the length of the horizontal guide channel to push the cut egg strands towards the bowl of artificial seawater. Transfer the sized egg strands to the artificial seawater. Repeat for all untangled egg strands.

### Straw-Strand Shuttle System (4S) (Fig. 5)

While performing this procedure wear appropriate personal protective equipment, including eye protection, laboratory coat, and when necessary insulated gloves. Hand fill with appropriate cryoprotectant the number of 0.5-mL French straws needed for an experiment. To hand fill the straws, insert a 200-µL pipet tip into the distal end of a 1-mL syringe. Insert the cotton end of a French straw into the wide end of the pipet tip. While supporting the straw and pipette tip, insert the open end of the straw into the cryoprotectant. Draw the cryoprotectant into the straw such that a 4.5-cm gap remains between the cotton and the leading edge of the cryoprotectant. This gap size is important and allows for insertion of three Strand Shuttles into the straw while ensuring presence of a small air gap between the level of the liquid and the Aligner Sealer. Remove the straw from the cryoprotectant and draw the cryoprotectant further into the straw so that the cotton is wetted, and the sealing powder is activated. Load straws into Straw Holders and allow the sealing powder in the cotton end of the straws to congeal for several minutes (1–2 min). Meanwhile, prepare several glass bowls of artificial seawater to keep the loaded shuttles wet during preparation and prepare several bowls of cryoprotectant for cryoprotectant equilibration.

Place a sized strand into each groove on a Strand Base. Align the strands by use of an Aligner Sealer to the correct distance from one edge of the Strand Base. Place a Strand Shuttle Cassette slot-side down on the Strand Base to capture one strand into each shuttle. Apply light pressure to the posterior side of each shuttle to ensure strand capture. The width of the strand slot (1.12 mm) on the shuttle is the perfect size to apply light pressure on the strand allowing the shuttle to capture the strand. Remove the Strand Shuttle Cassette from the Strand Base and place it in the bowl of artificial seawater. If any strands are not captured, replace the Strand Shuttle Cassette on the Strand Base and reapply light pressure. Repeat the strand capture procedure until enough Strand Shuttle Cassettes are filled.

Transfer the loaded Strand Shuttle Cassettes to bowls containing cryoprotectant and start a timer to track equilibration time. During equilibration and while supporting the Straw Holder, insert Strand Shuttle Cassettes into the straws and snap the shuttles off. Repeat this a total of three times per set of straws. After all shuttles are loaded, seal straws by inserting Aligner Sealer pegs into the open straw ends and snapping the sealers off. Continue equilibration at the appropriate temperature followed by appropriate cooling and storage. The present study did not evaluate cooling and storage.

Follow normal sample unloading procedures to unload the Strand Shuttle Cassettes from straws. Thaw straws at an appropriate temperature for an appropriate amount of time. Dry straws with a paper towel. Cut the Aligner Sealer-sealed end with scissors and, while holding the cut end over a container with artificial seawater, cut the cotton end to release the samples. Shuttles should exit the straw without need for further assistance. Shuttles that remain in the straw can be removed with gentle pressure generated with a syringe (setup as described in the straw filling section).

## Validation and characterization

To demonstrate the operation of the hardware and characterize its performance a time study was performed comparing egg mass preparation and packaging with and without the Strand Cryopreservation Shuttle System (SCSS). All data and R code are available at https://www.github.com/jackckoch/Strand-Cryopreservation-Shuttle-System. To evaluate strand preparation, four operators processed 10 egg masses characterized as having varying degrees of untangling complexity (e.g., easy, medium, or difficult). Untangling complexity was assessed by eye and was mostly based on egg mass compactness (Fig. 6).

**Fig. 5:**
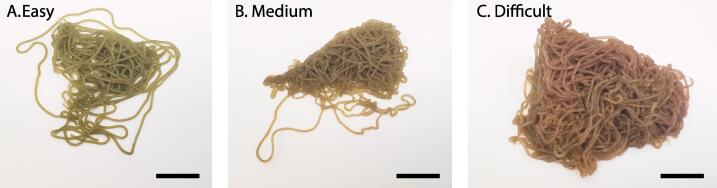
(Left) Simplified process map and (Right) timelapse images of hardware-augmented 4S method. Sketch of complete straw packed with three strand straw shuttles and sealed with an aligner sealer.

**Fig. 6:**
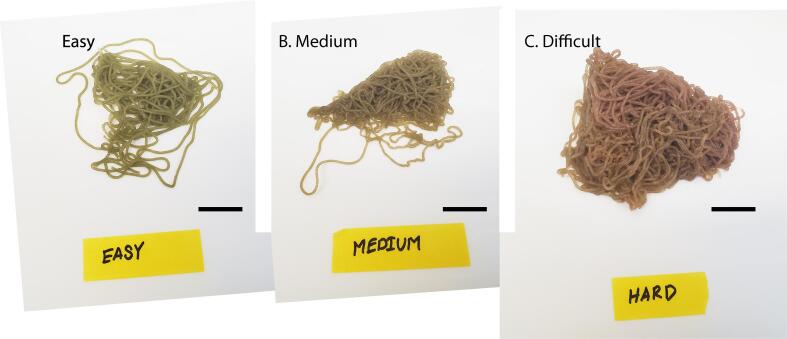
Images of *Aplysia* egg masses representative of untangling difficulty. (A) Easy, (B) Medium, and (C) Difficult. Scale bars = 19 mm.

Untangling operators had mixed levels of experience. Operators recorded the amount of time it took to untangle an egg mass and the resultant number of 1-cm egg strand pieces obtained during the subsequent cutting process. Sections of egg strands that were straight (no bends or kinks) or detached from the main egg mass were preferentially processed. Because deposited egg masses harden about 10 min after extrusion, bends and kinks were difficult to remove and were discarded or cut around during egg mass processing. In total, operators untangled enough egg mass to produce 2,042, 1-cm egg strands over 207 min or about 10, 1-cm strands per min. These data did not have equal variance and were not normally distributed. A Kruskal-Wallis rank sum test was used to evaluate differences in untangling efficiency based on untangling complexity (χ^2^ = 2.3036, df = 2). There was no significant difference (p = 0.32), in untangling efficiency based on the characterized complexity of the egg mass. Operators averaged 15.2 strands per min with masses rated “easy”, 10.6 strands per min with masses rated “medium”, and 5.5 strands min with masses rated “difficult”.

This highlights the opportunity to develop a device or technique in the future to capture straight strand sections while the animal is producing the egg strand. An alternative could be to dissolve the “adhesive” that cements egg masses together, but after egg strands have hardened (time varies by egg mass, personal communication, Phillip Gillette, NRA, 2023), curves and bends are difficult to straighten (personal observation). This technique would require rapid intervention after an egg strand had been produced which is not ideal based on current *Aplysia* culturing procedures at the NRA. A device that could be attached to an egg strand during deposition and wind 10-cm pieces around a spindle could be more practical.

**Fig. 7:**
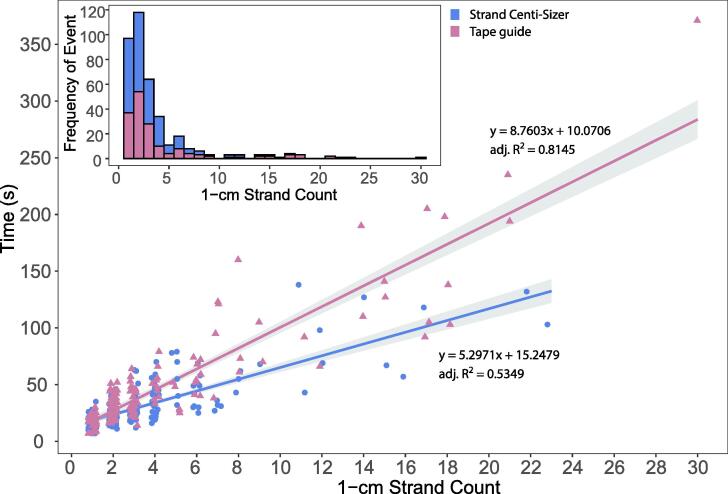
Time study of egg strand sizing from untangled egg masses. Each point represents an untangled egg strand piece that was sized (blue circles = Strand Centi-Sizer; pink triangles = tape guide). Linear models were fitted to the data for each sizing method (Strand Centi-Sizer: y = 5.2971x + 15.2479, adj. R^2^ = 0.5349, p < 0.001; tape guide: y = 8.7603x + 10.0706, adj. R^2^ = 0.8145, p < 0.001), with standard error of each model represented by the extent of the gray region around each line. The inset depicts the frequency that each number of 1-cm strand counts occurred, skewed heavily to the left.

Following egg mass untangling, strands were cut into 1-cm sections. This size was chosen for numerous reasons, and it is convenient for the NRA *Aplysia* culture practices as the target density for growth of planktonic *Aplysia* is 1 larva per mL [18] and a 1-cm strand contains an average of 1,500 to 2,000 larvae (personal communication, Michael Schmale and Phillip Gillette, NRA, 2021). For strand cutting, untangled strands from a single mass were split into two groups: sizing by use of a tape guide or sizing by use of a Strand Centi-Sizer (1-cm cut intervals). All 10 masses that were untangled previously were sectioned. Cutting operators (n = 4) had mixed levels of experience. Operators recorded the amount of time it took to cut each untangled strand and the resulting number of sized strand pieces. The timer started when a piece of strand was removed from the artificial seawater and stopped when the sized pieces were deposited into a bowl of artificial seawater. A linear model was fitted to each group of data based on the method used to size and cut strands. Operators using tape guides were able to produce 972, 1-cm pieces over 182 min or 5.3, 1-cm strands per min and operators using Strand Centi-Sizers were able to produce 979 1-cm egg strands over 161 min or 6.0 1-cm strands per min. This amounts to an average 12 % increase in efficiency when using the Strand Centi-Sizer to size strands or an additional 60 strands per hr per person (Fig. 7). The efficiency of this process increased as the number of sized pieces from a single strand increased (efficiency at scale). For example, the process efficiency was lower when a 2-cm egg strand was cut into 1-cm pieces compared to the process when a 20-cm egg strand was cut into 1-cm pieces. This further supports the need to develop a means of capturing straight egg strands during the egg strand laying process.

**Fig. 8:**
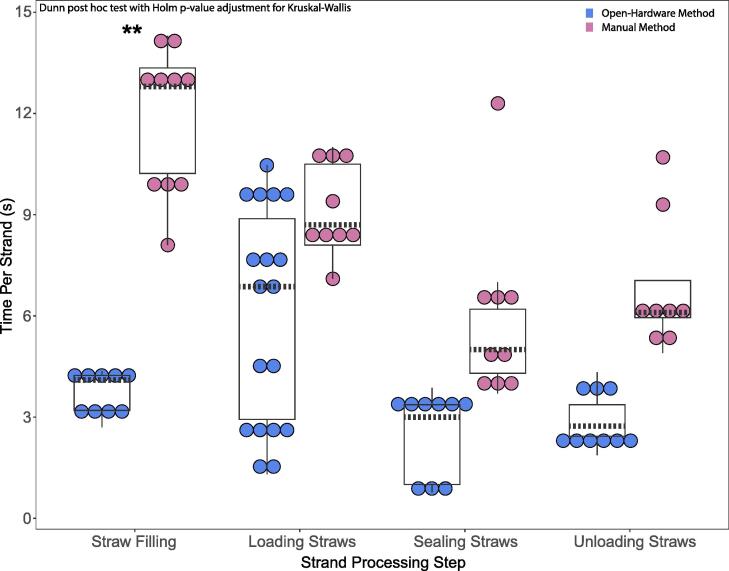
Time study of egg strand processing. Each point represents a replicate of 10 straws normalized to the number of strands per straw (blue circles = Straw-Strand Shuttle System, 3 strands per straw; pink circles = load-and-plunge method, 1 strand per straw). The median values of each group are indicated with dashed black bars, interquartile ranges indicated with black boxes and minimums and maximums indicated by vertical black lines. Comparisons were made using a Dunn post hoc test with Holm p-value adjustment for Kruskal-Wallis test (** indicates p < 0.001).

Sized pieces were packaged into 0.5-mL French straws and straws were sealed and unloaded. Cryopreservation cooling was not evaluated in the present study. Trials using the load-and-plunge method loaded one strand each in 10 straws, while trials using the 4S loaded three shuttles (three strands) each in 10 straws. Each trial was repeated nine times across two operators (n = 3 for operator 1, and n = 6 for operator 2). Operator 1 had a higher level of experience than operator 2 with the processing techniques. Operators timed four steps during each trial: straw filling, straw loading, straw sealing, and straw unloading (Fig. 8). Straw filling was accomplished using the same technique for all trials and was performed as described previously in operation instructions. The timer started when a straw was loaded onto the pipet-tipped syringe and stopped when the tenth straw was filled. During the load-and-plunge method, straw loading was performed as described previously in hardware description (Fig. 3). The timer started when a strand was removed from the bowl of artificial seawater and stopped when the tenth strand was plunged into the straw. Straws were individually sealed for 4–10 s by use of an UltraSeal21 (Minitube, Verona, Wisconsin) ultrasonic sealer. The timer started when the first straw was picked up and stopped when the tenth straw was sealed.

During the 4S method, straw loading was initially divided into two steps (shuttle loading and straw loading) but subsequently combined. In general, shuttle loading took about twice as long as straw loading. For shuttle loading, the timer started when a strand was removed from the artificial seawater and stopped when the cassette was placed into a bowl of artificial seawater. For straw loading, the timer started when a cassette was removed from the artificial seawater and stopped when the third cassette was snapped into the straws. Straws were loaded into a Straw Holder to align them for straw sealing. Ten straws were sealed simultaneously by inserting the Aligner Sealer sphere into the straws and snapping the pegs off. The timer started when the Straw Holder was picked up and stopped when the pegs were snapped off. Straw unloading was accomplished using the same technique for all trials and was performed to ensure that shuttles and strands would exit the straws without difficulty. Straw unloading was performed as described above in the operation instructions. The timer was started when the first straw end was cut and stopped when the shuttles or strand exited the tenth straw. The efficiency of this process was higher when accounting for the increased number of strands per straw (Fig. 8). For example, when the number of strands is not accounted for, the loading straws step of load-and-plunge method is on average faster (91 s) than the 4S method (181 s). Including more strands per straw increased storage space efficiency (e.g., 240 straws per standard daisy goblet [IMV, L’aigle, Basse-Normandie, France] = 720 egg strand pieces with the 4S method compared to 240 egg strand pieces with the load-and-plunge method).

These data for both methods had unequal variance and were not normally distributed. A Kruskal-Wallis rank sum test was used to evaluate differences between the methods (χ^2^ = 51.536, df = 7, p < 0.0001). A Dunn Kruskal-Wallis multiple comparison post hoc test with the Holm p-value adjustment was used to evaluate differences between each method for each step (e.g., straw filling, loading straws, sealing straws, unloading straws). Straw filling with for the 4S method was significantly faster than for the load-and-plunge method (Z = −4.27, adj. p < 0.001). There was no significant difference between the other steps (loading straws, sealing straws, unloading straws) for each method. An interesting follow-up comparison could compare the effects of operator experience, number of attempts, and order of method execution.

### Limitations

**Fig. 9:**
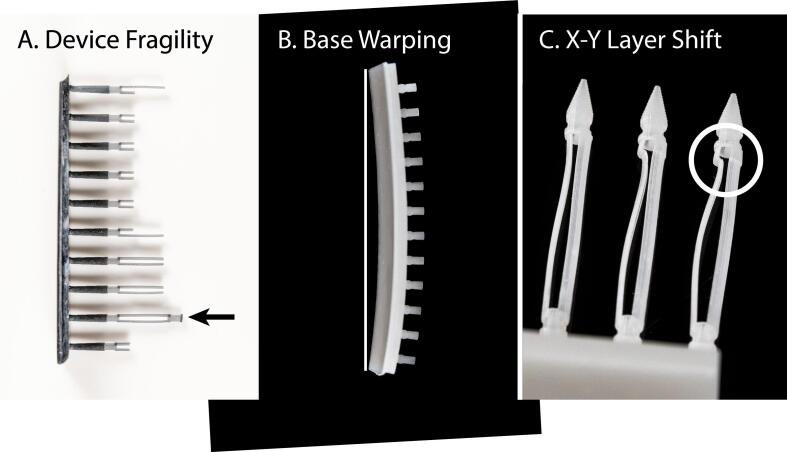
Images of several issues experienced with 3-D printing. (A) Device fragility especially of fine features. Black arrow indicates an undamaged feature. (B) Base warping. Vertical white line indicates where a flat base would have appeared. (C) X-Y layer shift. White circle indicates region of feature that experienced an X-Y layer shift, compromising the functionality of the device.

Several aspects of 3-D printing caused difficulty in strand processing. Part fragility, base warping, and layer shifts were the three most common issues, mostly in resin-printed components (Fig. 9). Part fragility was characterized by the fine features of the resin printed parts breaking during the printing or preparation process (Fig. 9A). The Strand Shuttle Cassettes were the most fragile components. Base warping was characterized by the base of the resin printed parts curving after curing and caused the alignment between the straws and the shuttles or sealers to be offset (Fig. 9B). The resin parts contained one third flexible resin which could have contributed to the base warping but also meant that the base could be slightly bent by hand during use with reduced likelihood of snapping. Base warping could be alleviated by a new base design and optimized curing. Layer shifts were typically a concern in resin printed parts but could occur in FFF parts and were characterized by an X-Y axis shift in multiple printed layers (Fig. 9C). A contributing factor to layer shifts was use of magnetic build plates and could be alleviated almost completely by use of a standard glass build plate (although there is a tradeoff between ease of removing prints and potential layer shifts). Some layer shifts caused no difficulty for strand processing but layer shifts that occurred while printing Strand Shuttle Cassettes often caused difficulty in loading the shuttles into the straws. If layer shifts occurred while printing Strand Shuttle Cassettes, the best course of action was to re-print the Strand Shuttle Cassettes. A straw loading operator noted that faulty Strand Shuttle Cassettes contributed to higher-than-average straw loading times. As such, a quality control step should be included for inspection and validation of parts after printing, in accordance with an overall quality management approach for establishment of cryopreservation programs for aquatic species [19].

### Improvements

Multiple improvements have been made to the system and are summarized in the present work. Time studies included herein utilized a previous version of the straw holder (v1.3.0). This version was resin printed, had no hinge or latching mechanism, and did not provide a friction grip on the straws. These features meant that the operator had to take care in supporting the straws and holder and often had difficulty keeping the straws aligned when sealing the straws. An improved version of the straw holder is included (v1.9.4) that was FFF printed to alleviate issues with base warping, included a hinge and latching mechanism, and provided a friction grip on the straws to ensure alignment and stability during loading and sealing. The 4S system could be further developed for many other applications including preservation of genetic resources from other organisms and ultra-rapid freezing (vitrification; e.g., [20], [21]). As such, the 4S is intended to provide a foundation for creating a novel vitrification system. The Strand Centi-Sizer could also be further developed for many other applications where a precise and guided cut needs to be made repeatedly. Overall, design for these devices could be improved by exploring alternative materials or combinations of materials to improve durability and ergonomics. Beyond the devices presented herein, an additional device that collects or winds egg strand (untangled) as it is extruded from the animal could improve this system of devices.

### Conclusions

Open hardware allows for the creation of affordable and customizable devices to address recurrent and novel needs in many applications and fields [14]. Open devices can be shared digitally and thus are a substantial improvement over manual, typically idiosyncratic, measures that limit reproducibility and scalable application. The switch from manual methods to open-hardware devices presented in this study provide improvements to several critical process factors such as time, money, people, and efficiency as well as increased standardization and reproducibility. In much-needed future development of germplasm repositories and cryopreservation pathways for aquatic species, open devices will be critical in enabling and scaling cryopreservation application in remote sites, areas with limited funding, and in research and commercial-scale environments [22]. This study presents a multiple-device integrated system that is designed for research and eventual direct application in cryopreservation pathways for the protection of *Aplysia* sea hare genetic resources, but the outcome of these devices is generalizable beyond *Aplysia*. Novel future applications include vitrification (e.g., ultra-rapid cooling to avoid ice crystal formation), enhanced sample preparation and handling, and augmented through integration of electronics and sensors [23], [24]. In addition, the power of open-hardware devices is not limited to the actual hardware but is extended by the possibilities for modification provided to other users and communities [25].

## Ethics statements

The policies and guidelines of the Institutional Animal Care and Use Committee that oversees LSUAC research are limited to vertebrate species, and as such are not applicable for invertebrates.

## CRediT authorship contribution statement

**Jack C. Koch:** Conceptualization, Methodology, Validation, Formal analysis, Investigation, Data curation, Writing – original draft, Writing – review & editing, Visualization, Supervision, Project administration. **Allyssa M. Oune:** Conceptualization, Methodology, Validation, Investigation, Writing – original draft, Writing – review & editing, Visualization. **Sarah Bodenstein:** Methodology, Validation, Formal analysis, Investigation, Writing – original draft, Writing – review & editing. **Terrence R. Tiersch:** Conceptualization, Writing – review & editing, Funding acquisition, Supervision, Project administration.

## Declaration of Competing Interest

The authors declare that they have no known competing financial interests or personal relationships that could have appeared to influence the work reported in this paper.
